# Improving GENCODE reference gene annotation using a high-stringency proteogenomics workflow

**DOI:** 10.1038/ncomms11778

**Published:** 2016-06-02

**Authors:** James C. Wright, Jonathan Mudge, Hendrik Weisser, Mitra P. Barzine, Jose M. Gonzalez, Alvis Brazma, Jyoti S. Choudhary, Jennifer Harrow

**Affiliations:** 1 Wellcome Trust Sanger Institute, Wellcome Genome Campus, Hinxton, Cambridge CB10 1SA, UK; 2 European Bioinformatics Institute, EMBL, Wellcome Genome Campus, Hinxton, Cambridge CB10 1SA, UK

## Abstract

Complete annotation of the human genome is indispensable for medical research. The GENCODE consortium strives to provide this, augmenting computational and experimental evidence with manual annotation. The rapidly developing field of proteogenomics provides evidence for the translation of genes into proteins and can be used to discover and refine gene models. However, for both the proteomics and annotation groups, there is a lack of guidelines for integrating this data. Here we report a stringent workflow for the interpretation of proteogenomic data that could be used by the annotation community to interpret novel proteogenomic evidence. Based on reprocessing of three large-scale publicly available human data sets, we show that a conservative approach, using stringent filtering is required to generate valid identifications. Evidence has been found supporting 16 novel protein-coding genes being added to GENCODE. Despite this many peptide identifications in pseudogenes cannot be annotated due to the absence of orthogonal supporting evidence.

## Abstract (web summary)

Identifying and annotating functional elements in the human genome remains a challenging but important task. Here the authors propose a priority annotation score to rank identifications and suggest how proteogenomics evidence can be interpreted and what additional information substantiates protein-coding potential for annotation.

## Graphical abstract

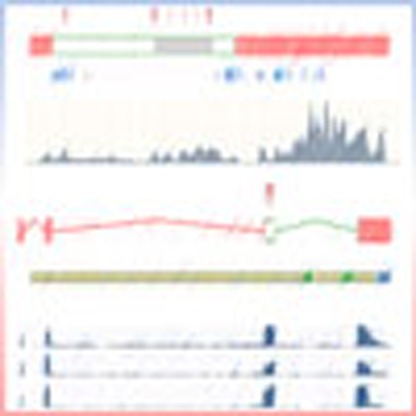

Biological and biomedical research relies on complete accurate and consistent annotation of genes and their products. However, identifying and annotating functional elements in the human genome is a persistent challenge. Despite intensive study, especially in identifying protein-coding genes, our understanding of the genome is far from complete, particularly with regard to non-coding RNAs, alternatively spliced transcripts and regulatory sequences. The GENCODE consortium is carrying out the annotation of all gene features in the reference human genome^1^. The annotation combines computational analysis, targeted experimental evidence and manual curation approaches to produce high-accuracy genome annotations for protein-coding genes, long non-coding RNAs (lncRNA) and pseudogenes. The reference gene set produced by GENCODE is the default gene set available in Ensembl.

A key goal of genome annotation is to describe a complete list of protein-coding genes. For many genes, confirmation of coding potential and the definition of the exact gene structure are not trivial^2^. Automated algorithms utilize comparative genomics methods such as phyloCSF^3^ to score transcript-coding potential while annotation groups incorporate antibody tagging^4^ and mass spectrometry^5^,^6^,^7^ proteogenomic experiments. GENCODE release (V22) contains only 19,814 protein-coding genes, decreasing from over 20,600 protein-coding loci in release V7. This fundamental refinement can mainly be attributed to improved comparative and proteomics analysis methods that have indicated transcripts with poor coding potential for removal from the reference gene set.^8^

High-quality peptides from large-scale proteomics experiments confirm coding potential, especially for genes and transcripts where there is little other supporting evidence^9^,^10^. Proteomics experiments only identify a fraction of detectable peptides. A peptide may not be detected if it is not amenable to the methods used or if the corresponding protein is not expressed in detectable quantities in the tissue type analysed. This means that while reliably identified peptides verify protein-coding potential, the lack of corresponding peptides does not prove a transcript is not translated. Despite this, the majority of protein-coding genes in the GENCODE annotation of the human genome are routinely identified with confident peptides in large-scale proteomics experiments^11^,^12^,^13^,^14^,^15^.

The increased scale of proteogenomics endeavours means that a higher false positive rate is inevitable. Recent reviews of the proteogenomics field have highlighted the challenge posed by false positives^16^,^17^. Target-decoy database searching is the most common approach for determining false positive identification rates, however, recent publications have pointed out some of the limitations of this method^18^,^19^,^20^,^21^. To minimize erroneous identifications, accurate estimation of error and conservative significance filtering criteria must be applied. While initial filtering by a false discovery rate (FDR), calculated based on decoy identifications, is a good starting point, novel identifications should be examined independently and assessed by posterior error probability (PEP) and *P* value. The use of tools such as Percolator can also correct for inaccuracies in FDR estimation^22^. Moreover, since many peptides are only detected occasionally, combining results from many experiments can improve proteome coverage and confidence of novel assignments. In addition, even with high confidence in peptide identifications alternative explanations such as sequence variants and contaminates must be considered. Proteogenomics experiments have been particularly useful for poorly annotated genomes, providing a powerful tool to validate new gene models^23^,^24^,^25^.

Proteogenomics analysis can generate large numbers of putative novel protein-coding gene identifications. However, there are no suitable guidelines for how this data should be treated so that it can be successfully incorporated into genome annotation. We present here criteria for the generation and interpretation of proteomics evidence for novel annotations. We describe a proteogenomic workflow encompassing a search database, and stringent filtering criteria to refine peptide assignments (Fig. 1). To assist annotators, we propose a priority annotation score to rank identifications. From the annotator's perspective, we discuss how proteogenomics evidence can be interpreted and what additional information substantiates protein-coding potential for annotation. In the presented work, we concentrate on the human genome by reanalysing human tissue data from the draft human proteome experiments, but these methodologies are applicable to any sequenced genome.

## Results

### Reanalysis of the human proteome

**Figure 1:**
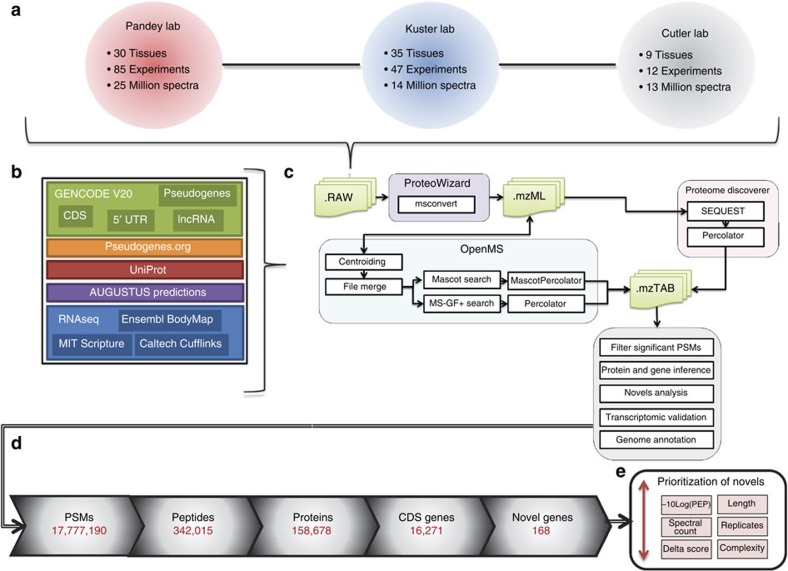
Proteogenomic analysis pipeline. (**a**) 4,917 raw files from the draft human proteome data sets^14^,^15^; containing 52,236,496 spectra comprising 61 different healthy human tissues were reprocessed. Non-normal tissue and cell lines may have compromised genomes, affecting expression of genes and displaying genome rearrangements and were not used here. (**b**) A bespoke protein sequence database was created containing 4,200,154 protein sequences. 270,908 of these redundantly mapped to known protein-coding genes and transcripts. This database comprised known protein sequences combined with translated RNAseq data and novel gene sequences (lncRNA, pseudogenes, 5′ UTR and novel gene predictions made using AUGUSTUS^39^). All isoleucine residues were replaced with leucine throughout the database to avoid complications caused by isobaric peptides. These sequences were concatenated with contaminants and decoy sequences to allow false discovery estimation. (**c**) Raw data was converted to mzML and searched in a proteogenomics workflow combining multiple mass spectrometry search engines and post-search evaluation tools. Results were filtered by peptide length, FDR, PEP and agreement between the multiple search algorithms. (**d**) About 34% of the spectra searched were significantly identified at a high-confidence level, collapsing to a smaller set of unique peptide sequences. Peptides were inferred into a redundant list of proteins, and mapped to a non-redundant set of known coding Ensembl (CDS) genes. Putative novel peptides were further filtered at an increased stringency. (**e**) A set of peptide features were combined in a score and used to rank novel identifications prior to manual annotation in GENCODE.

We have implemented the workflow shown in Fig. 1, and used it to reprocess over 52 million spectra from the human proteome experiments. This resulted in 17,777,190 significant peptide spectrum matches (PSMs) corresponding to 342,015 distinct peptide sequences longer than 7 amino acids (aa), representing 34% of the query spectra. PSMs were determined as significant using a 1% FDR and a 0.05 PEP cut-off. Significant PSMs were required to identify the same peptide with these criteria via multiple search algorithms. This equated to a final estimated PSM *q*-value of 0.0007 (0.07% FDR).

**Figure 2:**
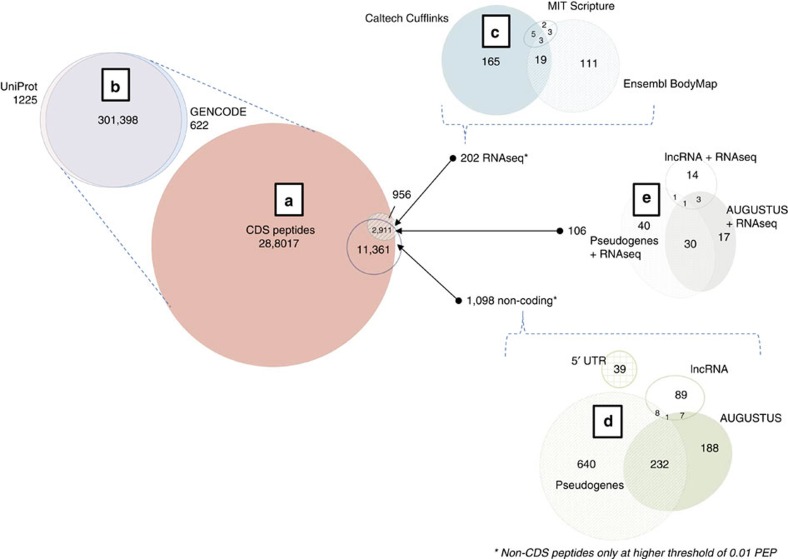
Identified peptide types. (**a**) Overlap of significant peptides between known coding sequences (GENCODE CDS and UniProt), RNAseq translations and novel proteins (5′ UTR, lncRNA, pseudogenes and AUGUSTUS predictions). (**b**) The overlap in known CDS peptides between GENCODE and UniProt. (**c**) Overlap in significant peptides between the three different RNAseq data sources. (**d**) Significant peptides matching non-CDS translated sequences. (**e**) Overlap between the annotated novel proteins and RNAseq data. All peptides have a minimum of seven amino acids and were identified by PSMs having an FDR <1% and posterior error probability (PEP) ≤0.05 based on two different search algorithms. Non-CDS peptides were reduced to a minimum PEP of 0.01 additionally passing a strict set of filtering criteria.

**Figure 3:**
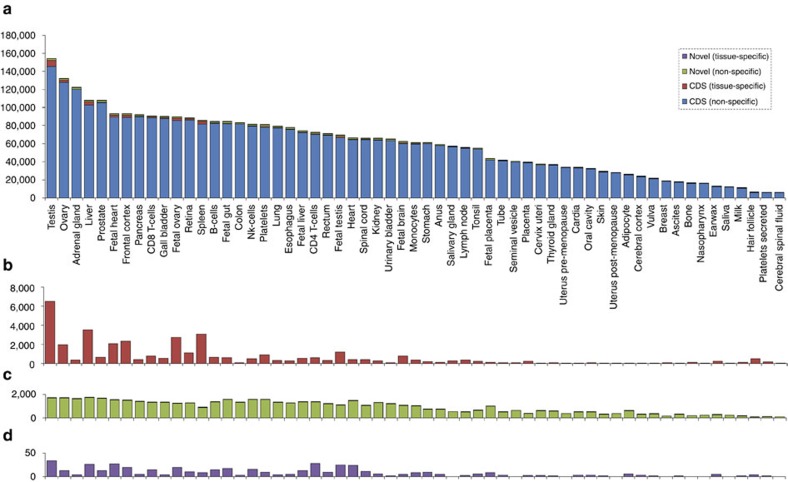
Tissue peptide specificity histograms. Plots showing the numbers of significant peptides identified for each tissue, depicted by tissue specificity and coding status (CDS/non-CDS). (**a**) This first plot shows the total number of significant peptides identified in each tissue, the majority of these being from known proteins expressed in multiple tissues. (**b**) This plot zooms in on the number of peptides from known proteins that are only observed in a single tissue. (**c**) This histogram shows only significant peptides mapped to novel proteins. (**d**) This final plot zooms down on those novel peptide identifications that are observed in a single tissue.

The distribution of identified peptides in the search space is shown in Fig. 2, with 99.5% matching known protein-coding sequences (CDS). About 0.6% of these were unique to either GENCODE or UniProt, highlighting the importance of considering multiple sequence annotation sources. These differences are due to independent database curation and annotation efforts. Figure 3 further breaks down the identified peptides by tissue, showing testis and ovary tissues to have the highest total number of peptide identifications. Testis also contained the most tissue-specific peptides, followed by liver, spleen, brain and foetal tissues. This observation suggests that these tissues have a significant number of uniquely expressed genes or alternative transcripts not found in other tissues. Other recent publications focusing on the analysis of Testis tissue have also found this to be true^26^.

Conducting protein inference and clustering to produce the shortest list of proteins that explain all significant peptides, we found evidence for 19,262 human proteins. For known proteins, we mapped these proteins to their Ensembl gene identifiers, finding evidence for 16,271 genes or clusters of genes with distinct peptides. A cluster of genes is defined as a set of genes, usually from the same family, which match one or more unique peptides specific to this set. Genes that only matched a subset of peptides from another gene were not counted in this number. About 13,852 genes were unambiguous identifications evidenced by multiple peptides and at least 1 uniquely identifying peptide. This is in reasonable agreement with the numbers of genes reported in previous studies using similar levels of stringency^13^. Further filtering by number of uniquely identifying peptides produces 11,606 and 10,740 genes with 2 and 3 unique peptides, respectively.

Among these genes, 7,716 transcripts were identified with unique peptides revealing 1,169 genes with evidence of alternative splicing. Further strict filtering increased the PEP threshold to 0.01, removed modified peptides, removed peptides with >30 aa and peptides with >2 missed cleavages. This additional filtering returned 32,497 gene-specific peptides and reduced the resulting genes with multiple different transcripts to 867. About 8,425 of the identified peptides crossed exon boundaries in these genes. About 1,869 transcripts mapped to these genes with 9,114 unique peptide mappings, and 2,956 of these peptides crossed exon boundaries. Testis again showed the highest amount of alternative transcription.

### Identifying novelty in the proteome

**Figure 4:**
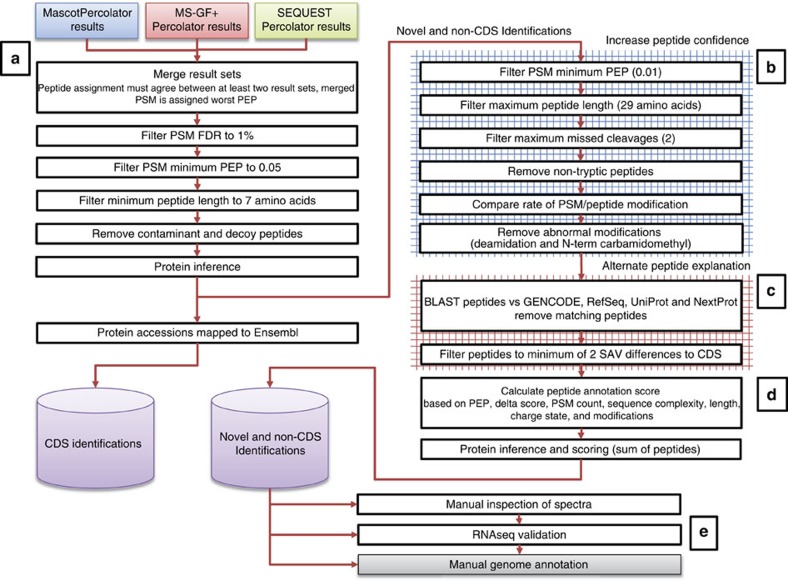
High-stringency peptide filtering regime. This workflow lays out the extensive filtering applied to search results before being passed on for genome annotation. (**a**) Results for multiple search engines are merged requiring PSMs to match between the different engines. The worst posterior error probability (PEP) between the matching search engines is carried forward. PSMs are then filtered by false discovery rate (FDR), PEP and peptide length. Contaminants are removed prior to protein inference. Peptides with CDS matches are mapped to Ensembl and stored while novel and non-CDS only peptides are further filtered. (**b**) Non-CDS peptides are first filtered to increase confidence in identification. (**c**) These peptides are then examined for alternative explanations or existing annotation. (**d**) The remaining non-CDS peptides are assigned a priority annotation score and inferred into novel or non-CDS genes. (**e**) The final set of ranked proteins undergoes manual inspection of spectra, validation against RNAseq data sets and is passed to manual annotators for review.

About 1,406 of the identified peptides did not match previously annotated coding regions within known protein-coding genes in GENCODE or UniProt. While these peptides could represent genuine novel CDS annotations, they could also be due to false positive assignments, or explicable as single nucleotide polymorphisms (SNPs) that lead to single amino-acid variants (SAVs). Genuine novel CDS annotations can take three forms: the addition of completely novel protein-coding genes where no previous annotation existed; the annotation of a CDS within a locus that was previously classified as an lncRNA or pseudogene; or the addition of novel CDS within pre-existing protein-coding genes (for example, the identification of additional coding exons). While the novel data is the most interesting in terms of GENCODE annotation, it is at the highest risk of false positive identifications. We examined these identifications and devised suitable filtering criteria and quality measures to produce a refined set of novel peptides for genome annotation (Fig. 4).

To generate higher confidence peptide identification, we increased our acceptance threshold to a minimum PEP of 0.01. Examination of the remaining spectra revealed that certain types of peptide commonly showed poor ion series coverage casting reasonable doubt on their assignments. As a consequence, long peptides >29 aa, peptides with >2 missed cleavages and semi-tryptic peptides were eliminated. In addition, we examined the incidence of post-translationally modified PSMs by comparing the enrichment of modification type in the novel data and the CDS data. These analyses found that deamidation and N-terminal carbamidomethylation were overrepresented among novel identifications (Supplementary Fig. 1). We have therefore disregarded all novel PSMs with these modifications. However, other modifications such as oxidation and acetylation showed similar proportions in both CDS and novel PSMs and have been retained, they only contribute a small portion (6%) of the novel PSMs.

The human genome has been the subject of intensive study and annotation for many years. Hence, the chances of a peptide being completely novel are small and an alternative explanation should be considered. There are several repositories of human genome annotation and these are being constantly updated with new annotations. Each novel peptide was blasted against the latest available GENCODE, RefSeq, NextProt and UniProt sequence database releases to identify peptides found in updated protein-coding annotations; 231 (7%) of the novel peptides were eliminated in this way. Also peptides containing SAVs will not match their corresponding protein sequence and can be misinterpreted as novel. To account for this, all novel peptides were required to be at least two amino acids different to any peptide from a known protein-coding gene.

The implemented peptide filtering regime should eliminate the majority of false positives. However, manual inspection of spectra is recommended for key peptides, especially those determined to provide evidence for new protein-coding regions. The final set of 650 highly confident novel PSMs mapped to 204 unique peptides and inferred 168 possible novel protein-coding annotations, 17 with multiple peptide evidence (Supplementary Dataset 1). In addition, any new gene annotations obtained were further validated by mapping tissue-specific RNAseq transcript data from the Uhlén study^27^ to the corresponding gene regions (Supplementary Fig. 4).

As part of this analysis, we proposed a priority annotation score for each novel peptide and protein. This score used peptide features suggested by expert annotators based on prior experience, targeting criteria that appeared to distinguish peptides that led to annotation from those that did not. The aim of this score was to rank novel peptides identifications, highlighting the protein and peptide mappings most likely to lead to new annotation. The peptide priority annotation score is based on various peptide features that are not normally considered in PSM scoring including the PEP, the number of PSMs observed, the number of replicate identifications, the delta scores between top and second rank assignments, peptide length and the amino-acid sequence complexity. The full formula used can be found in the online methods. The summed score of all unique peptides identifying a novel protein was then used to rank the putative novel protein identifications. In the context of our human proteogenomic analysis, this score was difficult to further optimize since, in spite of the speculative search space used, only a few novel genes were identified. This likely reflects the high quality of existing annotation for this reference genome. However, all filtered novel identifications were manually examined regardless of ranking, and we were thus able to demonstrate a good correlation between peptide score and the likelihood of annotation taking place (Supplementary Fig. 5). This priority annotation score is potentially more useful for annotators targeting non-model organism genomes, based on the presumption that such gene sets are less complete in terms of protein-coding content. However, we emphasize that the scoring system does not provide actual probability estimations; it should instead be used to rank potential proteins. Furthermore, while a score of ∼100 marks the point at which the annotation of a peptide became probable in this study, this is unlikely to be the same for studies on other genomes given the number of variables involved (such as the quality of genome sequence, and the size and nature of the proteomics data).

## Discussion

Manual annotation of the multi-peptide list, containing 17 prospective protein-coding regions, led to the addition of 7 protein-coding genes to GENCODE. In addition an eighth novel protein-coding region was created within the *INPP5F* protein-coding gene as part of an alternatively spliced transcript. Subsequently, 9 novel protein-coding genes were annotated in the list of 151 single-peptide identifications (Table 1). In total, our survey has led to the addition of 16 protein-coding genes to GENCODE: 8 based on lncRNA or RNAseq models, and 8 on pre-existing pseudogenes. The coding status of four of the pseudogenes—*MAGEB6P1, MYO15B* and two CENPV-related loci—were highlighted by Pandey *et al*.^16^, although *MYO15B* was previously regarded as a coding locus by UniProt. However, our *MYO15B* peptide is novel to this study and has supported the annotation of a CDS that is 1,534 aa longer than the UniProt entry. Two of the remaining 11 cases (HUMG00000150367 and HUMG00000158324) correspond to unreviewed entries in UniProt; the coding potential of the latter, in common with the CENPV-related loci, is also supported by data from PeptideAtlas^28^. Of the remaining cases, one represents our single agreement with the 404 translated lncRNAs proposed by Wilhelm *et al*.^15^ (ENST00000424358), leaving 9 protein-coding genes that represent novel discoveries.

**Figure 5:**
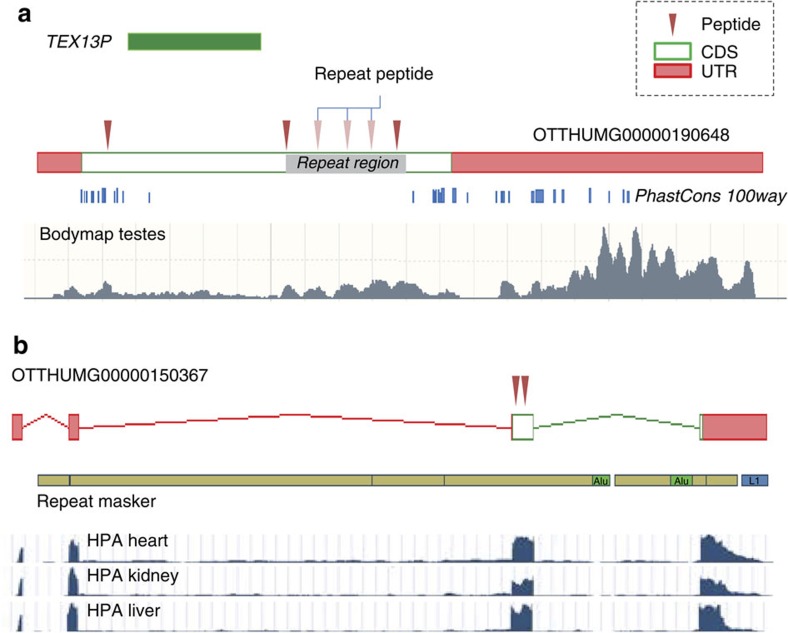
Novel protein-coding loci supported by multiple PSMs. (**a**) Protein-coding gene OTTHUMG00000190648 replaces processed pseudogene TEX13P on chromosome X. The latter had been based on an observed region of homology to TEX13A. The novel CDS is supported by four high-quality peptides unique to the locus (RNASEQ00000005460); one peptide aligns three times within a tandem repeat region. Transcription is apparently testis-specific in both the Illumina BodyMap and Human Protein Atlas RNAseq libraries; the BodyMap read coverage graph produced by Ensembl is shown below the model. The transcription start and end points of the novel structure are supported by CAGE data from the FANTOM5 project^55^ and polyAseq data from Derti *et al*.^31^ (not shown). PhastCons 100-way data are shown in blue (genome.ucsc.edu); while the CDS is conserved in mammals, there is length variation within the repetitive region. An orthologous gene model has been added to the mouse GENCODE annotation set as OTTMUSG00000054810. (**b**) lncRNA OTTHUMG00000150367 (ERVK1) has been reannotated as a protein-coding gene with a 109-aa CDS. This CDS is entirely found within repeat-masker sequence corresponding to the HERVK3, although the first exon is found within non-repeat sequence. RNAseq libraries indicate that the transcription of this locus is widespread, for example, found in all 27 Human Protein Atlas tissue RNAseq libraries generated by Fagerberg *et al*.^56^ as processed by Hezroni *et al*.^57^ (heart, kidney and liver coverage graphs are shown below the model), while the three high-quality PSMs generated here have been identified in 9 tissues. The most distant orthologue that can be confidently annotated is found in the rhesus macaque genome.

These annotations have resulted from a combination of the proteogenomic data presented here and the HAVANA manual annotation process, which incorporates next-generation transcriptomic data sets such as RNAseq^29^, CAGE^30^ and polyAseq^31^ that were not available during the genome-wide annotation phase of the GENCODE project. Certain pseudogenes, for example, were previously wrongly annotated since correct transcript structures could not be deduced, a problem confounded by 11 of the 16 novel protein-coding genes having single-exon CDS. Incorrect transcript structures can also prevent the identification of CDS based on conservation and paralogy. This is illustrated in Fig. 5a, which shows a protein-coding gene reconstructed on the site of a shorter pseudogene of *TEX13A*. RNAseq data is required to support the transcript structure, where testis-specific transcription is observed in agreement with the proteomics results, and the 714 aa CDS shows widespread conservation across mammalian genomes. In total, 15 of the 16 novel protein-coding genes either display mammalian conservation beyond primates or are putative members of known gene families. The outlier is shown in Fig. 5b, an endogenous retroviral element-associated CDS with conservation limited to certain primate genomes.

**Figure 6:**
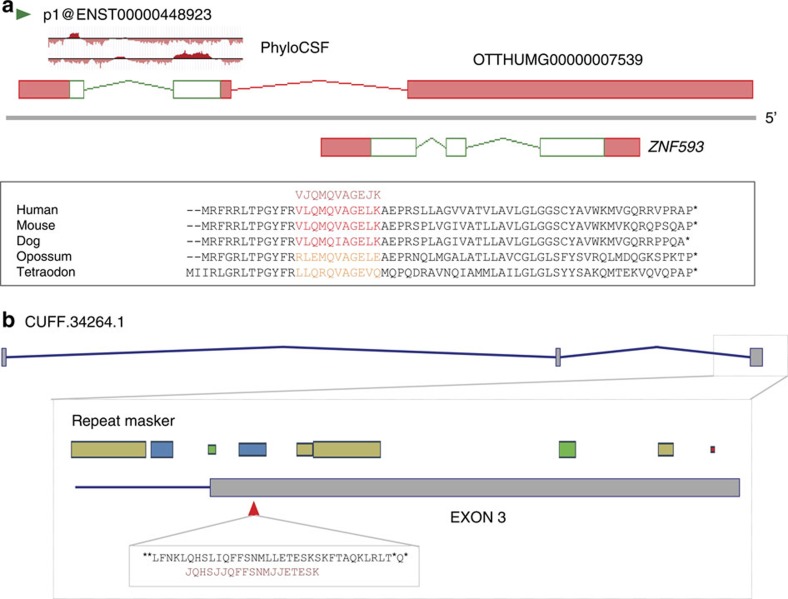
Identifying novel protein-coding regions based on single peptides. (**a**) A novel 63-aa CDS has been identified within OTTHUMG00000007539, previously lncRNA ENST00000407889. Alternatively spliced transcripts have been omitted for clarity. PhyloCSF plots are shown for the relevant reading frames^3^: the CDS is conserved in mammals, while a putative orthologue in the tetraodon genome is identified within an unplaced contig. The peptide (which spans the splice junction) was found only in retina. Strikingly, all transcriptomic data identified in each highlighted species is limited to eye tissues: human CAGE data from FANTOM5 (ref. 55; green triangle) identifies TSS within corneal and lens epithelial cells, vitreous humour and retina tissues; mouse CAGE data identifies TSS within eyeball tissue; the 6 splicing human ESTs identified are all derived from eye tissues, as are each of the 20 tetraodon mRNAs, 10 dog ESTs and 3 mouse mRNAs identified (not shown). (**b**) A 19-aa peptide is found within Cufflinks gene CUFF.34264.1, containing 3 exons spanning ∼175 kb of genomic sequence on chromosome 12. However, we do not observe support for these introns, the TSS or the transcript endpoint. The peptide falls within the final exon, which does not show evidence of conservation in PhastCons 100-way plots, and entirely within repeat-masker sequence (specifically an L1M6 element; www.repeatmasker.org). The ORF to which the peptide aligns does not contain a canonical initiation codon, and the distance between the flanking STOP codons is just 34 aa.

Despite the conservative and rigorous criteria presented here, 90% of our novel peptide identifications have not led to annotation, for two main reasons: either annotation did not make biological ‘sense' in a transcriptomic context, or there was an absence of orthogonal supporting evidence for the translation or transcription of the model. In the latter case, we note that numerous identifications were made solely within Cufflinks-derived RNAseq models; none of these led to protein-coding annotation, despite many of these peptides appearing to have good PSMs. In fact, few of these structures were seen to be supported by orthogonal transcript evidence, and so could not be added to GENCODE even as lncRNAs. Since proteogenomic surveys must be wary about alternate peptide explanations and the persistence of false positives, these non-annotations are therefore suggestive in this regard. Due to the difficulty in confirming peptide-to-protein inference, many of these novel peptide assignments may be correct but originating from a protein source not considered in our search space. Although the peptide data was essential in highlighting the putative CDS in each of these cases, it was the orthogonal data sets that ultimately led to annotation, in particular the observation of mammalian conservation. For illustration, Fig. 6 compares an 11 aa lncRNA-associated peptide (Fig. 6a) with a 19-aa peptide identified from an RNAseq model (Fig. 6b). The shorter peptide identifies a novel 63-aa CDS exhibiting strong vertebrate conservation, with striking eye-specific transcriptomic data matching the identified peptide. In contrast, the longer peptide is found 4 aa downstream of a STOP codon within a LINE transposon, in a region that shows no signature of protein-coding evolution. Indeed, we did not annotate any lineage-specific CDS in the single-peptide list, even when they could be linked to canonical initiation codons. We propose that lineage-specific CDS should only be annotated based on the presence of multiple, high-quality and manually validated peptides, unless other sources of experimentally derived supporting evidence are available.

Pseudogenes provide a particular interpretative challenge; such loci account for the majority of identified novel peptides. These loci differ from the pseudogenes converted to protein-coding genes previously discussed; they contain sequence disablements such as frameshifts or premature termination codons, whereas the latter are *bona fide* protein-coding genes previously mis-annotated. One could assume that pseudogene peptides are incorrect, given that these loci are traditionally thought of as being non-functional. Our initial concern was that peptides attributed to pseudogenes may actually derive from paralogous loci outside the search space, or from allelic variant forms of the parent genes. We found little evidence for these scenarios, although it is plausible that some pseudogene peptides could be explained by undocumented parental allelism, the presence of paralogous loci within reference assembly gaps, the existence of unappreciated copy number variation or retro-transposition events that are not fixed in the human lineage. These cases would be difficult to confirm without proteomic and transcriptomic experiments being conducted on paired samples.

It is interesting that many of the processed pseudogenes in our lists belong to families with dozens of copies, for example, the GAPDH family. All except one of these parental genes are described by Protein Atlas^32^ as producing a protein found at ‘high levels' in multiple tissues. It could be that highly transcribed genes produce more pseudogenes due to the increased chance of retro-transposition, raising the likelihood that one or more will gain the potential to be translated. Alternatively, it may simply be that higher levels of protein expression inevitably lead to an increase in the capture of dissonant spectra, or perhaps increase the likelihood of cryptic *in situ* modifications that are subsequently misinterpreted. Ultimately, it is clearly logical to be conservative in the interpretation of pseudogene peptide evidence. As for lineage-specific lncRNA CDS, pseudogene translation should only be considered for annotation where more than one high-quality unique peptide can be linked to an in-frame initiation codon. For such cases, a novel biotype ‘translated pseudogene' has been created in GENCODE; conversion to full-protein-coding gene status will only occur when the functional basis of the locus is experimentally established.

Proteomics has potential as a powerful tool for genome annotation, allowing validation of existing protein-coding annotations and discovery of novel coding regions. It offers substantial benefits for less refined genomes, although our identification of 17 novel coding regions demonstrates that mass spectrometry has an important role to play in human annotation. It is notable that despite the high levels of stringency and filtering used, the majority of peptides identified outside the known protein-CDS have not led to novel annotations, emphasizing a need for vigilance in interpreting such data sets and the need to handle pseudogenes identified by proteomics with care. Recent years have seen a rapid increase in the number of proteogenomics experiments published, many of which strive to obtain the largest coverage of peptides and novel identifications possible. The size of these data sets places a burden on annotators, as they strive to separate those genuine identifications from the inevitable set of spurious peptides. We believe that proteogenomics should take a ‘quality-over-quantity' approach, as exemplified by the integrated mass spectrometry and annotation workflow presented here. In addition, we propose a priority annotation score for ranking novel proteomics evidence. Although of limited benefit in this study, the higher scoring peptides did lead more frequently to new annotation, and we believe this would be of great value in non-reference genomes. Above all, proteomics data sets should not be interpreted in isolation, rather they should be considered as a single component of an annotation strategy that also includes high-throughput transcriptomic data coupled with comparative analyses.

## Methods

### Raw data sources

Three large, publicly available human tissue proteomics data sets were downloaded from the Internet. The first, containing 2,212 raw files, was generated by the Pandey lab at Johns Hopkins University^14^, and was downloaded from ProteomeXchange via the PRIDE^33^ repository (www.ebi.ac.uk/pride/archive/ accession PXD000561). These were higher-energy collisional dissociation (HCD) raw files, from Thermo Scientific Orbitrap instruments, comprising 85 fractionated experimental samples covering 30 different human adult and foetal tissues. The second data set was the Human BodyMap data generated by the Kuster lab at the Technische Universität München as part of their draft human proteome publication^15^, which was downloaded from ProteomicsDB (www.proteomicsdb.org accession PRDB000042). This data set consists of 1,087 HCD and collision-induced dissociation (CID) Thermo Scientific raw files from 48 experiments covering 36 different tissues. The final data set which was also used in the draft human proteome was generated by Paul Cutler at Roche Pharmaceuticals and originally deposited in PeptideAtlas^34^ in 2011. This data was also downloaded from ProteomicsDB (accession PRDB000012) and contains 1,618 CID Thermo Scientific raw files covering 10 different human tissues.

### Spectral processing

Each raw file was converted to the standard mzML format using the ProteoWizard (v3.0.6485) msconvert tool^35^. Following conversion, the data was processed with TOPP tools from OpenMS (pre-v2.0 development build) (Fig. 1)^36^. All spectra were centroided using the PeakPickerHiRes tool, and files from fractionated experiments were merged using the FileMerger tool (up to a maximum file size of 2 GB). A small number of the raw files appeared to contain no spectra and were not included in the conversion. The final set of 195 mzML files comprised 52,236,496 spectra.

### Sequence database creation and preparation for searching

A comprehensive human sequence database in FASTA format was created by combining six parts. These included, the complete human GRCh38 GENCODE v20 (ref. 37) CDS translated sequences; the UniProt^38^ human reference proteome from May 2014; common contaminant protein sequences downloaded from Max Planck Institute of Biochemistry (http://maxquant.org/contaminants.zip) and HLA sequences from (http://www.ebi.ac.uk/ipd/imgt/hla/download.html); a selection of non-coding gene sequences from GENCODE v20 including pseudogenes, lncRNA sequences and 5′ untranslated region sequences; novel sequences generated using the AUGUSTUS gene predictor^39^; an additional set of two-way consensus pseudogene predictions from Pseudogene.org (December 2013); and three-frame translated RNAseq transcript sequences. RNAseq transcript models were imported from three sources: Ensembl RNAseq models assembled using data from the Illumina Human BodyMap 2.0 project, which captures transcription in 16 human tissues (http://www.ebi.ac.uk/arrayexpress/experiments/E-MTAB-513); models generated by the Kellis lab at MIT using also Human BodyMap data and the Scripture software^40^; and models produced by Caltech and CSHL using RNAseq data from different ENCODE cell lines and the Cufflinks software^41^. GENCODE non-coding gene sequences, AUGUSTUS predictions, pseudogenes and RNAseq models overlapping GENCODE coding regions were filtered out from the final sequence database. For some of the databases the sequences needed to be translated into amino acids from nucleotide sequences; this was done using the EMBOSS (v6.6) six-pack tool^42^ to generate three-frame translations of the sequences, splitting stop codons into separate ORFs with a minimum length of 10 amino acids. Finally, a set of randomized decoy sequences of equal size to the target database was generated using the Mimic tool (https://github.com/percolator/mimic) and appended to the database. To account for isobaric peptides all isoleucine (I) residues within the database were replaced with leucine (L); after searching, leucine residues were always converted to the ambiguous code J. All protein accessions were formatted to include the source database, a unique identifier and if available a genomic locus. Supplementary Table 1 contains a summary of the sequence database components including the number of proteins, peptides and amino acids. In terms of distinct tryptic peptide sequences, the known coding portion of the database contained 787,587 peptides and the novel sequences provided an additional 4,211,835 peptides.

### Spectral identification and database search pipeline

Figure 1 describes the overall identification and analysis pipeline, including the OpenMS/TOPP-based workflow that was used to run the database searches for the three data sets. The TOPP tool MascotAdapterOnline was used to submit mzML files to a Mascot Server v2.4 (Matrix Science) cluster; the in-house developed TOPP tool MSGFPlusAdapter was used to run MS-GF+ v10089 (ref. 43) on the same files across a large computer cluster. Two wrappers were implemented to run MascotPercolator v2.08 (refs 44, 45) and the msgf2pin/Percolator v2.08-1 (ref. 46) tool combination to optimize and rescore the Mascot and MS-GF+ results, respectively. In addition, SEQUEST combined with Percolator^47^ was used to search the data in a Proteome Discoverer v1.4 (Thermo Scientific) workflow. All database searches were performed with a precursor tolerance of 10 p.p.m. and a fragment tolerance of 0.02 Da for HCD spectra and 0.5 Da for CID spectra. Up to three missed cleavages were allowed. The fixed modification carbamidomethyl (+57.0214) was specified for all cysteine residues. In addition, the following variable modifications were used in the searches: N-terminal acetylation (+42.01056), N-terminal carbamidomethyl (+57.0214), deamidation of asparagine and glutamine residues (+0.984), oxidation of methionine (+15.9949), and N-terminal conversion of glutamine and glutamic acid to pyro-glutamine (−17.0265, −18.0106). The multiple search results of this workflow were converted into mzTab formatted files and uploaded along with the mzML spectra and FASTA search database to the PRIDE repository (http://www.ebi.ac.uk/pride/archive/)^33^ under accession PXD002967.

### Initial results processing and filtering

Custom Perl scripts were used to parse, merge and filter the results of each search engine (Fig. 3). The results from the multiple search engines were first merged and filtered so that every PSM had the same identification in at least two of the three search engines. The highest (least confident) PEP value was retained in each case. The PSMs were then filtered keeping only matches with a *q*-value of ≤0.01 (1% FDR), a PEP of ≤0.05 and a peptide length of at least seven amino acids. PSMs matching contaminant or decoy sequences were also removed. Proteins were then inferred from the final list of peptides, taking a simple approach to cluster proteins that matched the same set of peptides, so that each protein cluster had at least one unique peptide. To calculate the number of genes identified by these proteins, we mapped GENCODE CDS and UniProt accessions back to Ensembl gene identifiers. Several protein clusters did not contain a GENCODE CDS nor a UniProt accession; these non-CDS protein identifications were separated and further filtered for genome annotation. Supplementary Fig. 4 displays the −10log(PEP) score distribution of CDS and novel PSMs.

### Non-CDS peptide analysis

Non-CDS identifications, which we define as peptides uniquely matching a single sequence not found in the GENCODE CDS, UniProt database nor the contaminant sequence databases were further filtered to an increased stringency. The filtering criteria included, a 0.01 PEP threshold, a maximum peptide length of 29 amino acids, only fully tryptic peptides, and a maximum of two missed cleavages. In addition after identifying an enrichment of deamidated and N-terminally carbamidomethylated PSMs in the non-CDS data set, PSMs so modified were also removed. Peptides were also checked to make sure that modifications were identified on the correct N-terminal residues in the case of pyroglutamate conversions. BLASTp^48^ with parameters optimized for short sequences was used to search an up-to-date combined GENCODE V22 (ref. 37), RefSeq V70 (ref. 49), NeXtProt (release 28 April 2015)^50^ and UniProt (release May 2015)^38^ sequence database. Any matches were removed from the data set. A Perl script was then used to search the GENCODE CDS removing peptides with less than two mismatches to known proteins.

### Priority annotation score

A score for each peptide was computed using a set of peptide features. The following equation describes the scoring for each protein based on the summed priority annotation scores of its distinct unambiguous peptides:


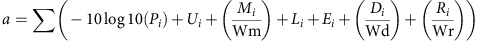


Where

*P*_*i*_ represents the best PEP obtained for each peptide, representing the confidence in this peptide.

*U*_*i*_ is the number of significant unmodified PSMs identifying each peptide, this increases the score of more abundant, repeatedly sampled peptides.

*M*_*i*_ is the number of modified PSMs significantly identifying each peptide, this is adjusted by Wm (initially set to 5), again this increases the score of more abundant peptides; however, the adjustment makes modified forms of the peptide less influential than unmodified PSMs.

*L*_*i*_ represents the peptide amino-acid length.

*D*_*i*_ is the delta score difference between 1st and 2nd ranked peptide spectrum assignments, adjusted by Wd (initially set to 10). This feature boosts the score where there is less ambiguity in sequence assignment, as this value can be large so it is divided by 10 to avoid it dominating the score.

*R*_*i*_ is the number of samples or replicates significantly finding each peptide this is adjusted by Wr (initially set to 5). This feature increases confidence in a novel peptide and reduces the chance of it originating from a variant sequence. In this study, there are over 100 samples included, hence we applied a weighting to prevent this feature overly influencing the final score.

*E*_*i*_ is the entropy for the peptide sequence calculated using information theory using this formula:


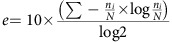


Where

*n*_*i*_ is the number of occurrences of each amino-acid type in the peptide and *N* is the length of the peptide. This feature increases the scores of more complex peptides.

The Wm, Wd and Wr weightings were initially set to values that scale the peptide features to the same magnitude and avoid any feature overly biasing the score. For use beyond this study these values can be optimized, as well as additional features added to suit the type of experimental data and annotation evidence required. Supplementary Fig. 5 shows a histogram of the resulting scores for peptides that led to novel annotations and those that were rejected on manual inspection of the genomic loci.

### RNAseq analysis and validation of novel genes

After retrieval of the publicly available raw data of the ArrayExpress data set with the accession number E-MTAB-2816 (ref. 27), the RNAseq libraries were analysed with iRAP using TopHat2 (ref. 51) to map reads to the reference genome (GRCh38.p3) and HTSeq-count^52^ to assign reads to the Ensembl release 76 gene annotation^53^ using default parameters. Reads Per Kilobase of transcript per Million (RPKM) values were then calculated with the internal function provided by iRAP. For each tissue, gene expression levels were calculated by first taking the mean of the technical replicates before calculating the mean of the biological ones. When the VEGA IDs of the genes had corresponding ENSEMBL 76 gene IDs, those IDs were directly used to assess the gene expression. When this was not the case, the coordinates of the genes were retrieved from the latest VEGA release and then the windows of interest were plotted on merged bam files (one bam per tissue) obtained from TopHat2. The Supplementary Dataset 1 includes the number of paired reads that have been counted for each novel gene on the merged bam files normalized by the number of initial samples for each tissue.

### Manual genome annotation

Manual annotation was performed according to the HAVANA criteria as defined for the GENCODE project^37^. A novel ‘logic tree' was used to allow annotation decisions to be made consistently; see Supplementary Fig. 3. Furthermore, a tblastn query was performed for each peptide on the primary list (see main text) to check whether related sequences found outside the search space could provide alternative explanations for the match; this was also done for those peptides on the secondary list seen to highlight putative coding regions. For pseudogene-associated matches on the primary list, dbSNP^54^ was queried to investigate whether the PSM could also belong to a variant form of the parent locus.

### Data availability

All search results along with the spectra and sequence database are available in the PRIDE repository (http://www.ebi.ac.uk/pride/archive/) under accession PXD002967. The authors declare that all other data supporting the findings of this study are available within the article and its Supplementary Information files.

## Additional information

**How to cite this article**: Wright, J. C. *et al*. Improving GENCODE reference gene annotation using a high-stringency proteogenomics workflow. *Nat. Commun.* 7:11778 doi: 10.1038/ncomms11778 (2016).

## Supplementary Material

Supplementary Information Supplementary Figures 1 - 5 and Supplementary Table 1

Supplementary Dataset 1 Novel Identifications. This table, made up of four sheets, contains a lists of inferred novel (non-CDS) proteins, peptides, PSMs and the final set of validated novel protein coding genes. Included in the protein tables are the match genomic loci, GENCODE VEGA identifiers, annotation notes, peptides matched, and tissues in which these peptides were seen. Additionally these tables also highlight which of these proteins have any further supporting proteomic evidence in the January 2016 release of PeptideAtlas. The table also contains the results from mapping tissue specific transcriptomic RNAseq data to the same locus. An RPKM above 0.75 is considered to show significant transcript expression at these loci. The peptide table gives more detail on individual peptides, including annotation score, best PEP, number of modified and unmodified PSMs, modification types, number of samples significantly identifying peptide and the list of tissues for these samples. The final sheet contains a list of the significant PSMs identifying the non-CDS peptides. This table shows the identification and scoring for the multiple search engines, the spectrum mzML ID, any modifications found in the spectra, and spectral charge state.

## Figures and Tables

**Table 1:** Novel GENCODE protein-coding annotations.

Vega ID	Protein model(s) matched	Original biotype	Gene description	#Unique peptides	Score	CDS size	Chr
OTTHUMG00000021534	AUGUSTUS	Pseudogene	Gene similar to CENPV	11	1,616.05	287	x
OTTHUMG00000191517	AUGUSTUS	Pseudogene	Gene similar to CENPV	9	1,397.35	272	x
OTTHUMG00000024197	RNAseq	Pseudogene	MOAP/PNMA-like gene	3	597.06	647	x
OTTHUMG00000190648	RNAseq	Pseudogene	Gene similar to TEX13A	3	518.91	714	x
OTTHUMG00000150367	lncRNA	lncRNA	HERV-related sequence	3	466.01	109	19
OTTHUMG00000032333	lncRNA	lncRNA	Unknown	2	373.14	71	20
OTTHUMG00000188075	Pseudogene, RNAseq, AUGUSTUS	Pseudogene	Cancer/testis (C/T) gene	2	308.09	324	x
OTTHUMG00000021284	Pseudogene, AUGUSTUS	Pseudogene	MAGEB6P1	1	229.67	407	x
OTTHUMG00000191553	RNAseq, AUGUSTUS	Nothing	Weak homology to LBH	1	217.25	108	14
OTTHUMG00000067448	RNAseq	Pseudogene	MOAP/PNMA-like gene	1	215.47	578	x
OTTHUMG00000179794	Pseudogene	Pseudogene	Myosin XVB	1	209.93	3,064	17
OTTHUMG00000022468	lncRNA	lncRNA	Weak homology to SMIM10	1	208.01	78	x
OTTHUMG00000007539	lncRNA	lncRNA	Unknown	1	128.43	63	1
OTTHUMG00000167731	lncRNA	lncRNA	Unknown	1	109.10	59	11
OTTHUMG00000158324	AUGUSTUS	lncRNA	Zinc-finger like gene	1	96.87	203	7
OTTHUMG00000191600	AUGUSTUS	Nothing	Unknown	1	89.64	177	1
**OTTHUMG00000019158**	**5′ UTR**	**Protein-coding**	**INPP5F**	**2**	**194.26**	**593**	**10**

CDS, coding sequences; UTR, untranslated region.

Based on the peptide evidence discovered in this study combined with additional orthogonal evidence, 17 annotations have been added or updated in the GENCODE gene set. The single addition within a previously existing protein-coding gene is highlighted in bold at the bottom of the table. The ‘Vega ID' identifies the updated annotation in the latest release, while the ‘Protein Models Matched' lists the search database sequence types matched by the peptides. The ‘Original Biotype' represents previous annotation at each locus and the ‘Gene Description' column shows known gene name or gene homology. Genes are described as ‘unknown' where no homology to known protein families or functional domains could be detected. The final columns show the number of unique peptides matching each protein model, the summed priority annotation score of these peptides, the length of the new CDS, and the chromosome on which this gene is located. A more complete version of this table can be found in Supplementary Dataset 1.
