# Supplementary material for: Improving GENCODE reference gene annotation using a high-stringency proteogenomics workflow
Source: Nat Commun. 2016 Jun 2;7:11778. doi: 10.1038/ncomms11778 (PMC4895710; doi:10.1038/ncomms11778)
Supplement: Supplementary Information — Supplementary Figures 1 - 5 and Supplementary Table 1 [file ncomms11778-s1.pdf]

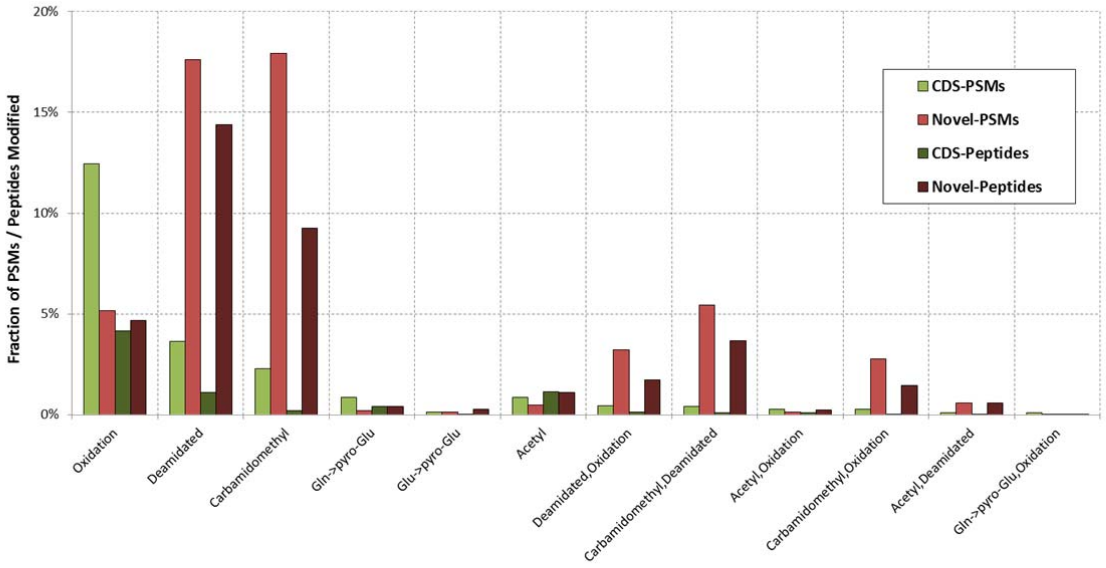

Supplementary figure 1: PSM and Peptide Modification Rate Comparison between Known CDS Proteins and Novels. This plot shows the rate of occurrence of deamidation, oxidation, acetylation, N-terminal carbamidomethylation, and pyro-Glu formation in the subsets of the PSM and peptide results. This demonstrates a clear bias for deamidation and N-terminal carbamidomethylation in the novel identifications. Based on this analysis we removed PSMs with these modifications from the final novel analysis.

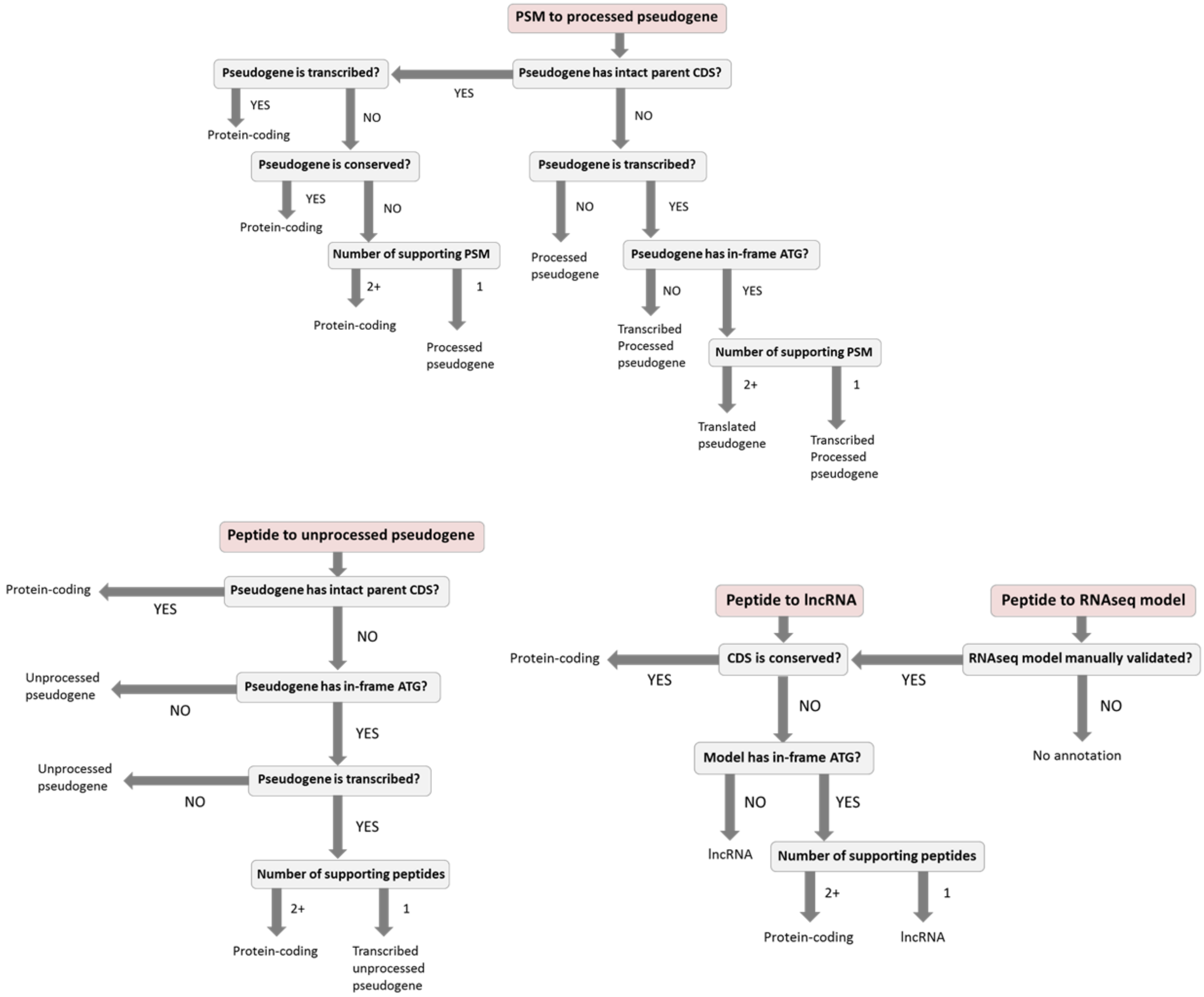

Supplementary figure 2: Decision Trees for the Annotation of Potential Novel Protein-coding Loci, Incorporating Mass Spectrometry Data. This logic is now used by the HAVANA team, and it has formed the basis for the analysis presented in this work. Only the core decisions are included, and this workflow should be considered as overlaid on top of the full annotation guidelines of the HAVANA project. Furthermore, in each scenario the supporting peptide identifications will be reappraised as outlined in the Methods section; additional matches will be sought outside the original search space, and for pseudogenes variation will be examined within the parent locus. For pseudogenes, an examination of the transcriptional potential of the locus is a key part of the decision-making process. At the present time, HAVANA judge pseudogene transcription solely based on Sanger-sequenced evidence sets, i.e. cDNAs and ESTs. RNAseq datasets are not currently appraised due to concerns about the potential for the mis-mapping of short reads between paralogous loci. However, we anticipate that RNAseq datasets will become vital adjudicators of pseudogene transcription in the near future as read lengths increase, e.g. based on the PacBio sequencing platform. The pseudogene CDS is defined as 'intact' with regard to the parent locus where the initiation and termination codons of the parent are found and the reading frame maintained. A pseudogene or lncRNA-associated CDS is regarded as 'conserved' when an orthologous CDS is found in other species, i.e. incorporating syntenic conservation. For human

annotation, HAVANA considers sequence conservation (including read-frame conservation) to be highly informative when it is displayed across a range of non-primate genomes. However, conservation may also be inferred within the primate lineage alone, provided supportive sequence data is available from a range of new world and old world monkey species. Conservation limited to ape genomes is not regarded as informative. In each scenario an ‘in-frame ATG’ is classed as an ATG found upstream of the peptide in the same reading frame, prior to a termination codon. In other words, the ATG could plausibly be used as an initiation codon to produce a translation that could explain the existence of the peptide. Our work found a single example of a unitary pseudogene (MYO15B), defined as a pseudogene that has vigorously defined protein-coding orthologues in other mammalian genomes. The annotation of mass spectrometry data for such cases essentially follows the decision tree for unprocessed pseudogenes, with ‘unitary pseudogene’ replacing the ‘unprocessed pseudogene’ output as where appropriate.

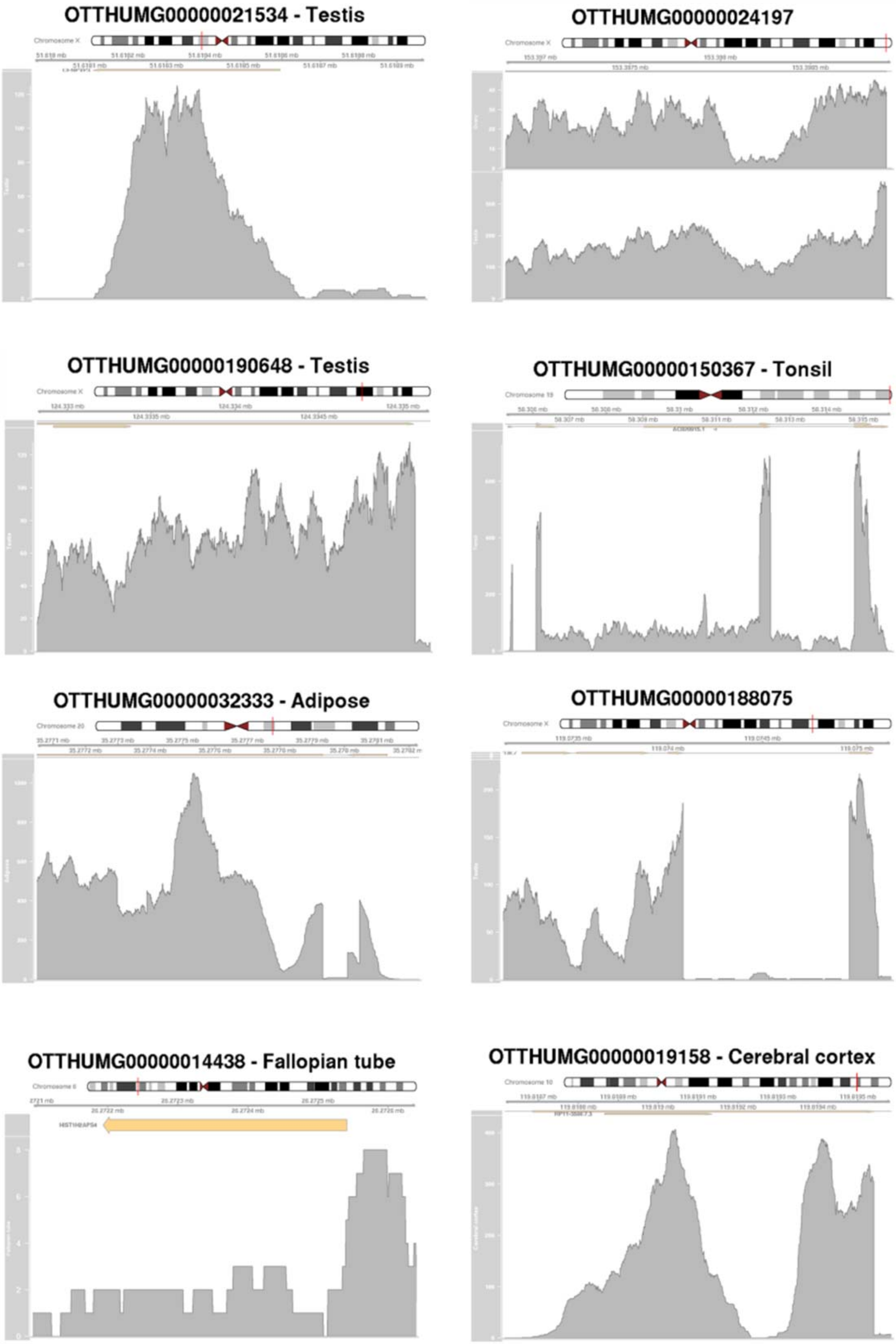
OTTHUMG00000021534 - Testis
OTTHUMG00000024197
OTTHUMG00000190648 - Testis
OTTHUMG00000150367 - Tonsil
OTTHUMG00000032333 - Adipose
OTTHUMG00000188075
OTTHUMG00000014438 - Fallopian tube
OTTHUMG00000019158 - Cerebral cortex

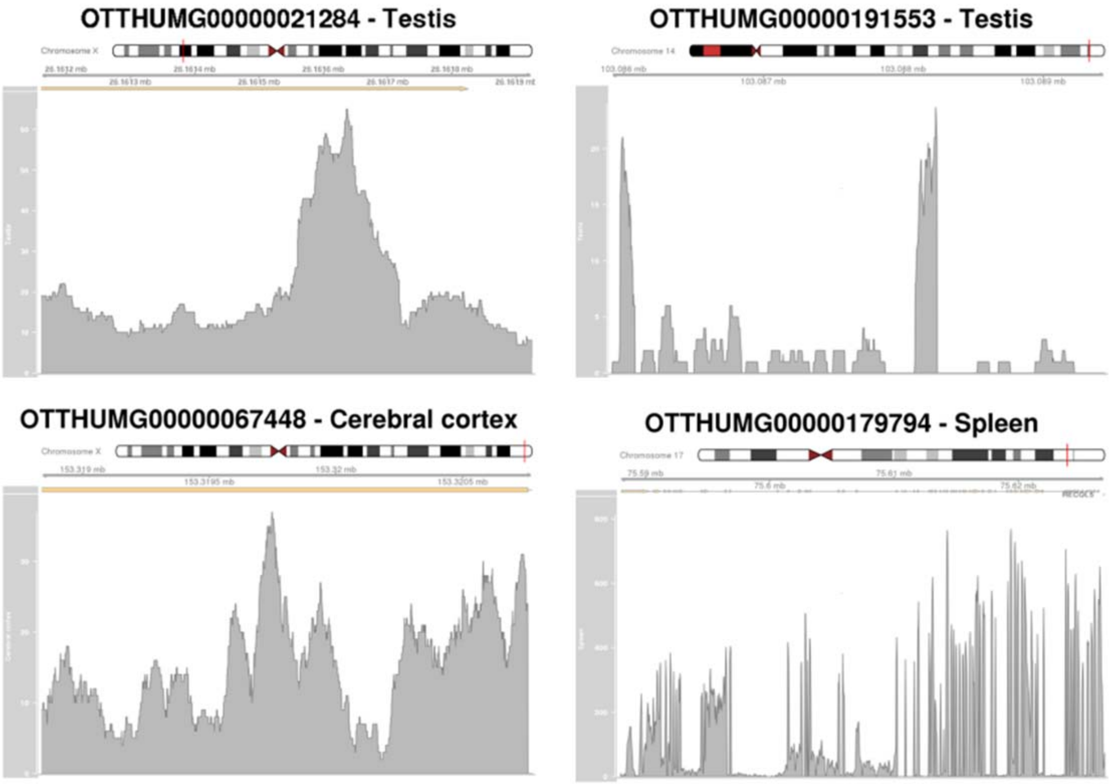
OTTHUMG00000021284 - Testis
OTTHUMG00000191553 - Testis
OTTHUMG00000067448 - Cerebral cortex
OTTHUMG00000179794 - Spleen

69

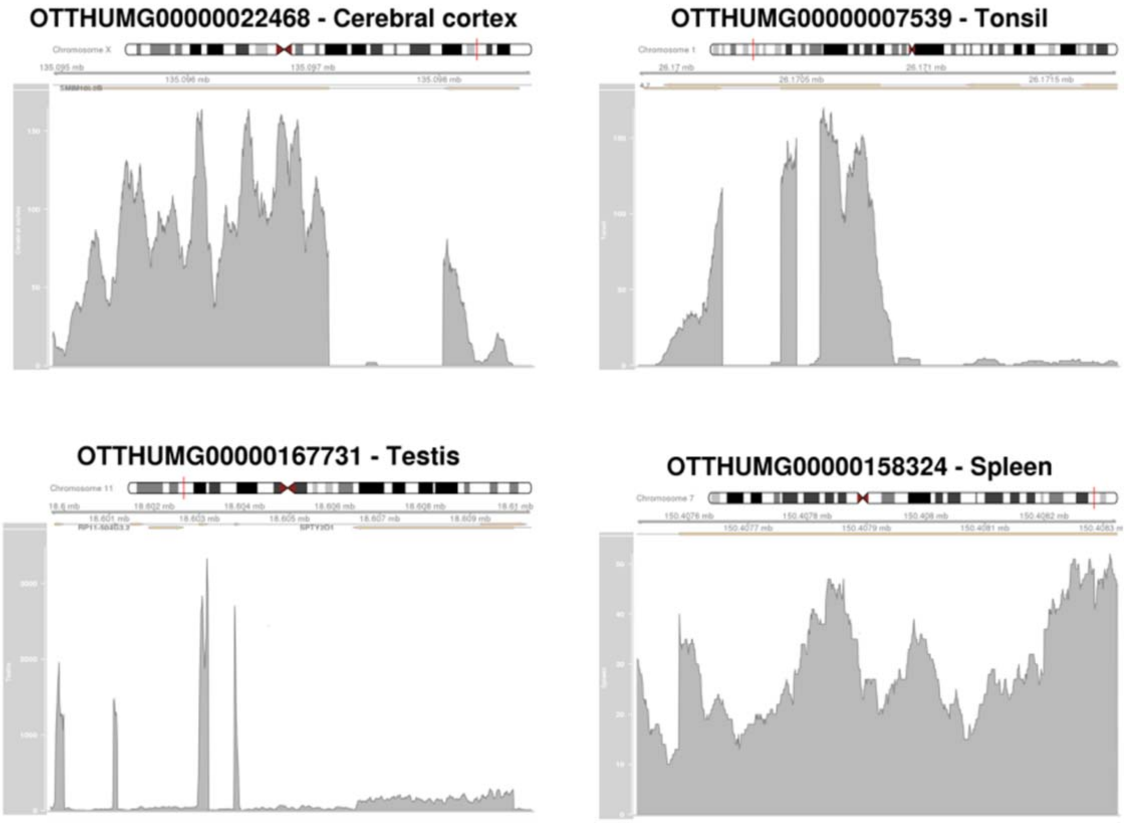
OTTHUMG00000022468 - Cerebral cortex
OTTHUMG00000007539 - Tonsil
OTTHUMG00000167731 - Testis
OTTHUMG00000158324 - Spleen

70

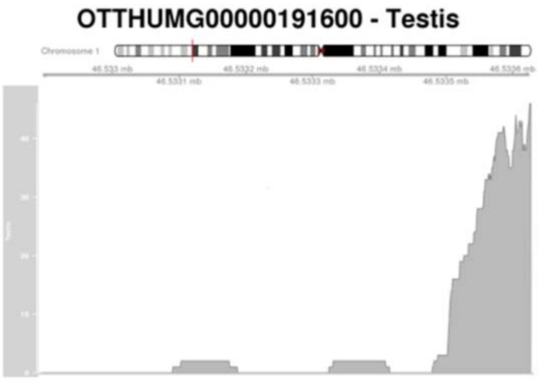

Supplementary figure 3: Tissue Specific Transcript Coverage Plots for Novel Protein Coding Genes. These plots depict tissue specific mapping of transcript data from ArrayExpress dataset E-MTAB-2816 [30], against each of the novel protein coding regions discovered in this study. The x-axis of each plot corresponds to the highlighted region of the genome in which the novel protein in located. The y-axis shows the number of paired reads mapping at this loci.

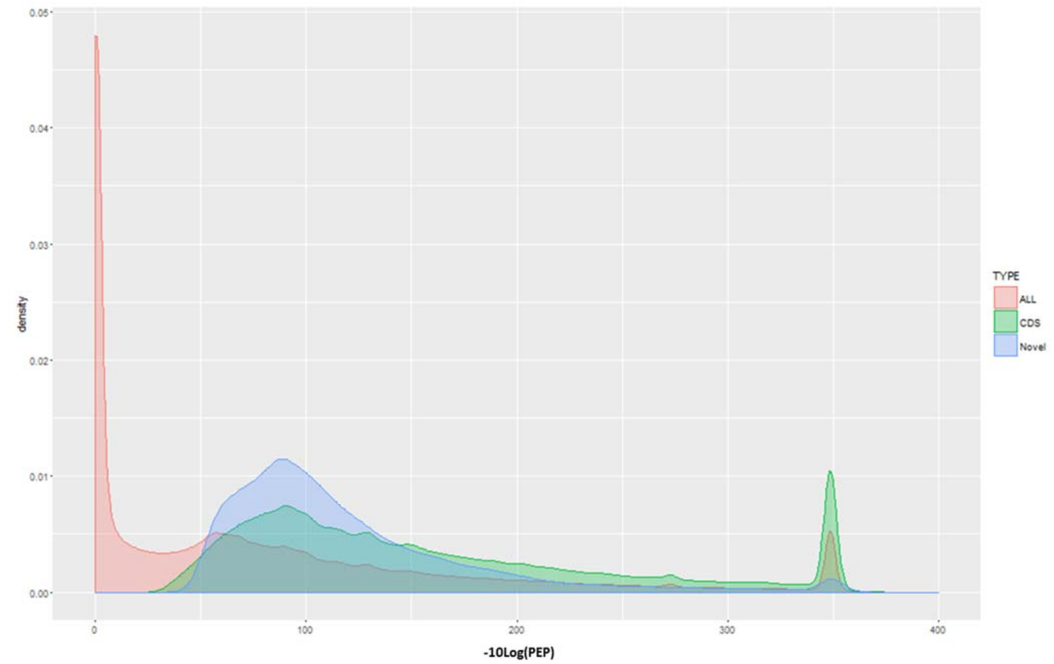

Supplementary figure 4: PSM Density Plot - This plot depicts the density distribution of PSMs based upon their -10* Log (Posterior Error Probability) values. The overall distribution is shown in red, the PSMs matching known coding sequences are in green and the PSMs identified as novel are shown in blue. Note that a score of 13 represents a PEP of about 0.05 and 20 represents a PEP of 0.01. Nearly all the novel and the majority of the CDS identifications are above a score of 50 which represents a PEP of 0.00001. There is a peak in the score at around 350 (PEP = 10^{-35}) we believe this is to be an artefact for the Percolation where the probability of a PSM being incorrect is very close to 0.

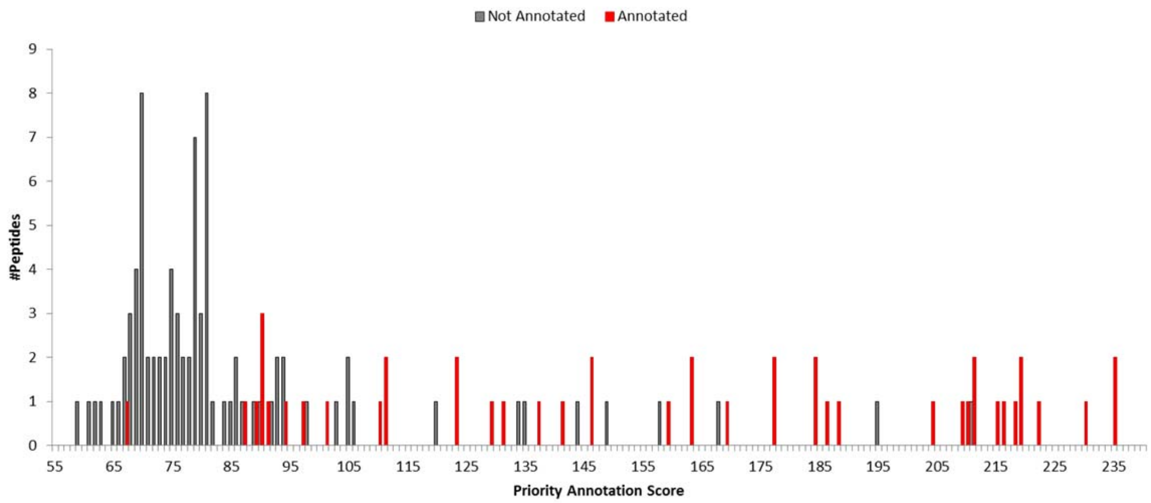

Supplementary figure 5: Peptide Priority Annotation Score Histogram – The x-axis of this plot represents the binned priority annotation score of novel peptides identified in this study, and the y-axis is the number of unique peptides in each scoring bin. The novel peptides that did not lead to a new GENCODE annotation are shown in grey and the peptides which have given rise to novel protein coding genes are shown in red. Peptides mapping to pseudogenes have been excluded due to the confounding and ambiguous nature of their annotation. A trend can be seen in this histogram where peptides that achieved a greater priority annotation score are much more likely to lead to a novel annotation after manual genome inspection and incorporation of further evidence streams. It should also be noted that the very low scoring peptides that did lead to annotation are from multi-peptide proteins which contained additional higher scoring peptides.

Supplemental dataset 1: Novel Identifications. This table, made up of four sheets, contains a lists of inferred novel (non-CDS) proteins, peptides, PSMs and the final set of validated novel protein coding genes. Included in the protein tables are the match genomic loci, GENCODE VEGA identifiers, annotation notes, peptides matched, and tissues in which these peptides were seen. Additionally these tables also highlight which of these proteins have any further supporting proteomic evidence in the January 2016 release of PeptideAtlas. The table also contains the results from mapping tissue specific transcriptomic RNAseq data to the same locus. An RPKM above 0.75 is considered to show significant transcript expression at these loci. The peptide table gives more detail on individual peptides, including annotation score, best PEP, number of modified and unmodified PSMs, modification types, number of samples significantly identifying peptide and the list of tissues for these samples. The final sheet contains a list of the significant PSMs identifying the non-CDS peptides. This table shows the identification and scoring for the multiple search engines, the spectrum mzML ID, any modifications found in the spectra, and spectral charge state.

Name	Description	Genome	NA Sequences	Proteins / ORFs	AminoAcids
Caltech Cufflinks	RNAseq transcripts	GRCh37	21789	2629390	74931806
UniProt	UniProt Complete Human Proteome	na	na	88708	35125351
Gencode CDS	GENCODE 20 Protein Coding Transcripts	GRCh38	na	93246	34827847
Gencode lncRNA	Long non-coding RNA genes	GRCh38	24460	704224	20081370
Gencode 5'UTR	5'UTR sequences for GENCODE CDS genes	GRCh38	59348	255141	7470191
Gencode PseudoGenes	GENCODE 20 Pseudogenes	GRCh38	10444	206225	6422183
Yale Pseudogenes	Pseudogene.org Sequences	GRCh37	6011	126160	3674056
Mit Scripture BodyMap	RNAseq transcripts	GRCh37	12206	73432	1812832
Ensembl BodyMap	RNAseq data from 16 different tissues	GRCh38	na	10280	1515295
AUGUSTUS	Additional gene predictions	GRCh38	na	6317	1250673
Contaminates	Standard list of contaminate proteins	na	na	247	130626
Decoy Database	Shuffled target sequences	na	na	4193370	187242230

Supplemental table 1: Search Database Components. This table summarises the sequence sources used to create the sequence databases searched using mass spectrometry data. Several of the non-protein coding and RNAseq transcripts were 3 frame translated and divided at stop codons into open reading frames ORFs.
